# A case of trazodone induced prolonged hypogeusia

**DOI:** 10.1192/j.eurpsy.2022.1856

**Published:** 2022-09-01

**Authors:** R. Tekdemir, A. Kandeğer

**Affiliations:** 1 Atatürk Chest Diseases and Thoracic Surgery Training and Research Hospital, Department Of Psychiatry, Ankara, Turkey; 2 Selçuk University, Psychiatry, Konya, Turkey

**Keywords:** trazodone, hypogeusia, side effect, oral cavite

## Abstract

**Introduction:**

Plenty of antidepressants have been reported to induce unpleasant tastes and/or odors as well as altered chemosensations when administered alone or in combination with other medications. Trazodone induced hypogeusia (decreased taste sensation) is a rare side effect. In this report, we would like to present a male patient with with hypogeusia after trazodona use and persisting for 3 months after the drug was discontinued will be discussed.

**Objectives:**

A 52-year-old male, Trazodone 50 mg/day was started 4 months ago due to difficulty in falling asleep. On the 25th day of her daily treatment, her sense of taste began to decrease and gradually became more severe. So he stopped his treatment and he applied to the internal medicine and neurology polyclinics. Routine blood tests were within normal limits. To rule out the possibility of covid 19, 2 pcr tests were done and it was found negative. No recommendations other than chewing gum. The patient applied to the psychiatry polyclinic with the complaint of decreased taste sensation that in the 3rd month of his complaints.

**Methods:**

CASE REPORT

**Results:**

CASE REPORT

**Conclusions:**

Chemosensory side effects due to drugs are frequently seen in the elderly and in polypharmacy. It is usually accompanied by a decrease in salivary secretion. It resolves shortly after the causative drug(s) are stopped. It is important that our patient is middle-aged, does not have additional medical diseases and does not use drugs, and his complaints continue for 3 months after the stopped of Trazodone.

**Disclosure:**

No significant relationships.

